# Interferon-gamma as adjunctive immunotherapy for invasive fungal infections: a case series

**DOI:** 10.1186/1471-2334-14-166

**Published:** 2014-03-26

**Authors:** Corine E Delsing, Mark S Gresnigt, Jenneke Leentjens, Frank Preijers, Florence Allantaz Frager, Matthijs Kox, Guillaume Monneret, Fabienne Venet, Chantal P Bleeker-Rovers, Frank L van de Veerdonk, Peter Pickkers, Alexandre Pachot, Bart Jan Kullberg, Mihai G Netea

**Affiliations:** 1 Department of Internal Medicine, Division of Experimental Internal Medicine, Radboud University Medical Center, Nijmegen, The Netherlands; 2 Department of Intensive Care Medicine, Radboud University Medical Center, Nijmegen, The Netherlands; 3 Department of Anaesthesiology, Radboud University Medical Center, Nijmegen, The Netherlands; 4 Department of Laboratory Medicine, Laboratory of Hematology, Radboud University Medical Center, Nijmegen, The Netherlands; 5 Joint Unit « Sepsis » Hospices Civils de Lyon–bioMérieux, Hôpital Edouard Herriot, Lyon, France; 6 Department of Internal Medicine (463), Radboud University Nijmegen Medical Center, P.O. Box 9101, 6500 HB Nijmegen, The Netherlands

**Keywords:** Immunotherapy, Interferon-gamma, Candidemia, Aspergillosis

## Abstract

**Background:**

Invasive fungal infections are very severe infections associated with high mortality rates, despite the availability of new classes of antifungal agents. Based on pathophysiological mechanisms and limited pre-clinical and clinical data, adjunctive immune-stimulatory therapy with interferon-gamma (IFN-γ) may represent a promising candidate to improve outcome of invasive fungal infections by enhancing host defence mechanisms.

**Methods:**

In this open-label, prospective case series, we describe eight patients with invasive *Candida* and/or *Aspergillus* infections who were treated with recombinant IFN-γ (rIFN-γ, 100 μg s.c., thrice a week) for 2 weeks in addition to standard antifungal therapy.

**Results:**

Recombinant IFN-γ treatment in patients with invasive *Candida* and/or *Aspergillus* infections partially restored immune function, as characterized by an increased HLA-DR expression in those patients with a baseline expression below 50%, and an enhanced capacity of leukocytes from treated patients to produce proinflammatory cytokines involved in antifungal defence.

**Conclusions:**

The present study provides evidence that adjunctive immunotherapy with IFN-γ can restore immune function in fungal sepsis patients, warranting future clinical studies to assess its potential clinical benefit.

**Trial registration:**

ClinicalTrials.gov - NCT01270490

## Background

The incidence of fungal infections is steadily increasing in the last years due to invasive medical diagnosis and immunosuppressive treatment modalities. Despite development of new classes of antifungal agents [1], the invasive fungal infections remain associated with unacceptable high mortality rates and represent a major cause of death worldwide [2-7]. The emergence of significant resistance to the currently available antifungal therapies emphasizes the need for novel approaches to treat invasive fungal infections [8,9]. Invasive fungal infection are most commonly observed in individuals with immune defects or a compromised immune system, and the number of these patients is steadily increasing [10]. Therefore, adjunctive immunotherapy to improve host defence is an attractive strategy to improve the outcome of patients with disseminated fungal infections.

In the past decade, major progress in the understanding of anti-fungal host responses has enabled the development of a number of novel molecular and cell-based immunotherapeutic approaches for invasive fungal infections [11]. Although invasive candidiasis and aspergillosis are rather different in their pathogenesis, the major protective host response against both fungi is the effective induction of Th1 and IFN-γ responses [12-16]. The Th1 cytokine response activates effector phagocytic cells that kill the fungus [17]. Interestingly, Th1 immunity against *A. fumigatus* was demonstrated to be cross-protective against *C. albicans*[18].

Interferon-gamma (IFN-γ), the prototype Th1 cytokine, promotes Th1 differentiation and skews the immune response towards a protective Th1 phenotype [19]. As such, it has been implicated as a treatment option in (invasive) fungal infections [20,21]. Moreover, limited evidence suggests that recombinant IFN-γ (rIFN-γ) has a beneficial effect on the outcome of fungal infections in patients with chronic granulomatous disease (CGD) [22], HIV [23-25], leukemia [26,27], and in patients receiving organ transplants [28]. However, it has not been investigated whether rIFN-γ actually enhances the immune response in these patients to explain these beneficial clinical effects.

In this report we describe a series of patients with invasive *Candida* and/or *Aspergillus* infections in whom we investigated the effects of treatment with rIFN-γ on the host innate and adaptive immune responses.

## Methods

### Patients and treatment

**Figure 1:**
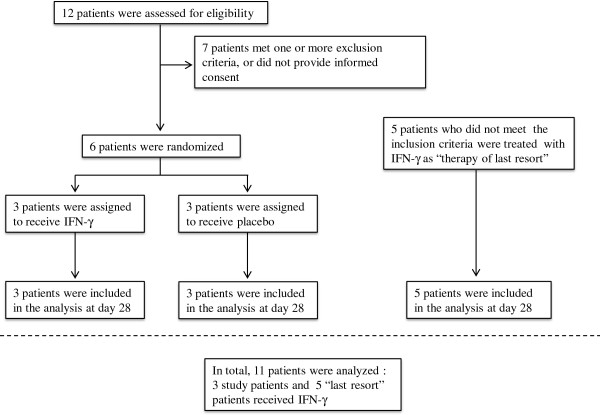
**Screening, randomization, and follow-up of the study patients.** The principal investigator was immediately notified when *Candida spp*. were cultured in blood. With at least one systemic inflammatory response syndrome (SIRS) symptom present in the 24 hours prior to blood culture withdrawal, and administration of systemic antifungal therapy < 72 hours, patients were deemed eligible for the ‘IFN-γ as an adjunctive treatment for candidemia’ pilot-study. In addition, 5 patients not meeting inclusion criteria but who were also treated with rIFN-γ as a therapy of last resort, were included in analysis.

To assess the feasibility and preliminary efficacy of IFN-γ in combination with anidulafungin for the treatment of candidemia, a single-centre, prospective, randomized open-label pilot (Phase IIIb) study was conducted. This study was registered at ClinicalTrials.gov (NCT01270490) and approved by the local ethics committee of the Radboud University Medical Center. Due to slower than anticipated enrollment rates (from August 2010 until March 2013, only 12 patients could be screened, of which 6 were eligible and provided informed consent [Figure 1]), the study was terminated early. However, during this period, several other patients presented with invasive fungal infections which had an insufficient response to standard antifungal therapy. Although these patients did not meet the inclusion criteria (i.e. presenting with one or more positive cultures of blood or normally sterile tissue growing *Candida* spp.), they were deemed to benefit from adjunctive immunotherapy as “therapy of last resort” as decided by the attending physician. Within the parameters of standard clinical care these patients were treated according to the same protocol as the patients enrolled in the study, and were therefore included in the present case series. All patients with a history of documented epileptic seizures, pre-existent severe renal impairment (creatinine clearance <30/mL/min) or severe liver failure (defined as a spontaneously increased prothrombin time) were excluded. After obtaining informed consent, eight patients (3 study patients, 5 last resort patients) were treated with rIFN-γ (Immukine, Boehringer Ingelheim, 50 μg/m^2^ body surface, subcutaneously, three times a week) in addition to standard antifungal therapy as recommended by national and international treatment guidelines [29,30]. Three patients who were included in the Phase IIIb *Candida* pilot-study were assigned to the control group and did not receive rIFN-γ.

### Blood sampling

Plasma, serum and whole blood specimens were collected at baseline (BL) and serially after the start of antifungal therapy (days 1, 2, 3, 7, 14 and 28). Blood cultures were performed as part of routine care.

### Leukocyte populations and surface HLA-DR expression

Heparin anticoagulated blood was stored at 4°C immediately after withdrawal and analyzed by flow cytometry. To determine the extent of immune suppression, HLA-DR expression was determined by calculating % HLA-DR-positive cells and HLA-DR mean fluorescence intensity (MFI) within CD14^+^ cells and various lymphocyte subsets within CD45^+^ leukocytes^+^ (see Additional file 1 and Additional file 2: Figure S1 for details and a representative flow diagram). Lymphocyte subsets were defined as: T-cells (CD45^+^CD3^+^), T-helper cells (Th, CD45^+^CD3^+^CD4^+^), cytotoxic T-cells (Tc, CD45^+^CD3^+^CD8^+^), B-cells (CD45^+^CD19^+^), and NK-cells (CD45^+^CD3^-^CD56^+^). Subset counts were calculated by multiplying the percentage of gated cells by the total lymphocyte count. Patients with <50% HLA-DR positive monocytes at baseline were considered to exhibit immune paralysis. This threshold of 50% is well below the lower bound of the 99% confidence interval obtained in healthy volunteers in an earlier study of our group using the same methodology in the same laboratory [31]. Therefore mHLA-DR expression levels below 50% are likely to represent immunoparalysis.

### Cytokine assays

Venous blood was drawn into 10 mL EDTA tubes, after which peripheral blood mononuclear cells (PBMCs) were isolated as described previously [32]. In short, blood was diluted in phosphate buffered saline (PBS) (1:1) and fractions were separated by Ficoll (Ficoll-Paque Plus, GE healthcare, Zeist, The Netherlands) density gradient centrifugation. Cells were washed twice with PBS and resuspended in RPMI-1640+ (RPMI-1640 Dutch modification supplemented with 10 μg/mL gentamicin, 10 mM L-glutamine, and 10 mM pyruvate) (Gibco, Invitrogen, Breda, The Netherlands). The PBMCs were counted using a particle counter (Beckmann Coulter, Woerden, The Netherlands) and were plated in 96 well round-bottom plates (Corning, NY, USA) at a final concentration of 2,5×10^6^/mL, in a total volume of 200 μL. The PBMCs were stimulated for 24 hours, 48 hours, and 7 days with medium alone, or medium containing *E. coli* lipopolysaccharide (LPS; 10 ng/mL), phytohaemaglutinnin (PHA; 10 μg/ml), heat-inactivated *Candida albicans* blastoconidia (1×10^6^/ml) or heat-inactivated *Candida albicans* hyphae (derived from 1×10^6^/m conidia). After stimulation, cell culture supernatant was collected and stored at -20°C. When all samples were collected, cytokines were measured using commercially available ELISAs (R&D Systems, MN, USA and Sanquin, Amsterdam, The Netherlands) according to the protocols supplied by the manufacturer. Ex-vivo production of cytokines was assessed at timepoints at which their production has been shown to peak [33]. Monocyte derived cytokines such as Interleukin (IL)-1β and tumour necrosis factor (TNF)α were measured in culture supernatants of 24 hour cultures, IL-10 was measured in culture supernatants of 48 hour cultures. T-cell derived cytokines IL-17 and IL-22 were measured in culture supernatants of 7 day cultures.

### Statistical analysis

In view of the small sample size, normality of distribution was not assumed. Comparisons of baseline with follow up time points were made using Wilcoxons signed rank test (within-group comparisons, 2 groups). A p-value of <0.05 was considered statistically significant. Data are expressed as means and standard error of the mean. Calculations and statistical analyses were performed using GraphPad Prism v 5.0 (GraphPad Software, San Diego, CA, USA).

## Results

### Patient characteristics

The patients treated with rIFN-γ (5 men, 3 women) had a median age of 49.5 [IQR 28.5-68.8] years. The three female patients in the control group were 36, 51 and 73 years old. Clinical characteristics of the participants are listed in Table 1. Of the 6 patients included in the pilot study, three patients had a positive blood culture for *C. albicans*, two patients for *C. glabrata*, and one patient for *C. tropicalis*. During randomization, the three patients with *C. albicans* cultures were assigned to rIFN-γ treatment, whereas the two patients with *C. glabrata* and one with *C. tropicalis* cultures were assigned to the control group. However, no pathophysiological evidence currently exists to suggest that rIFN-γ therapy would have a different effect on the immune system in case of albicans vs. non-albicans *Candida* infections. Of the other 5 patients treated with rIFN-γ as therapy of last resort, one patient had proven acute aspergillosis, one had probable acute aspergillosis, and one probable chronic aspergillosis according to the EORTC/MSG criteria [34]. One patient had a positive blood culture for *C. tropicalis* associated with osteomyelitis. This patient developed new suspected lesions on positron emission tomography-computed tomography (PET-CT) while receiving antifungal treatment. In another patient, CT-scan revealed progression of suspected hepatic *Candida* lesions during antifungal treatment. All patients included suffered some degree of immunosuppression: the 6 patients with positive blood cultures for *Candida spp*. had impaired physical barriers due to the presence of indwelling venous catheters (for the need of recurrent blood sampling or total parental nutrition), or an implantable cardioverter-defribillator (ICD) lead. The patient with progression of suspected hepatic *Candida* lesions on the CT-scan, and one patient with proven acute aspergillosis (diagnosed by isolation of *A. fumigatus* from lung tissue biopsies) were immunocompromised because of (therapy for) acute myeloid leukaemia. Another patient with acute invasive aspergillosis (*A. fumigatus* isolated from BAL fluid) received immunosuppressive therapy (prednisone and azathioprine) for sarcoidosis and suffered from a co-infection with *Mycobacterium genavense* localized in the bone marrow. A third patient developed chronic pulmonary aspergillosis (diagnosed by consistent CT abnormalities with cavitation and culture of *A. fumigatus* from BAL fluid) after radiotherapy for lung carcinoma. Only two patients, both with acute aspergillosis, were admitted to the Intensive Care Unit to receive organ supportive therapy (mechanical ventilation and hemodynamic support).

**Table 1:** Summary of clinical characteristics of all patients with invasive fungal infections

**IFN-γ treated patients**										
**Age**	**Pathogens**	**n=**	**Site of nfection**	**n=**	**Underlying illness**	**n=**	**Antifungal therapy**	**n=**	**Outcome**	**n=**
49.6 ± SD19.8	*C. albicans*	3	Candidaemia	2	Stem cell transplantation for AML	1	Anidulafungin	1	Cured without further infectious complications	2
**BMI**	*Candida spp*	2	Candidaemia Endocarditis	1	Sarcoidosis treated with prednisone and azathioprin	1	Fluconazole	1	Lost to follow up after discharge from hospital	1
22.9 ± SD6.9	*A. fumigatus*	2	Pulmonary aspergilllosis	3	First remission induction chemotherapy for AML	1	L-AMB + Voriconazole	1	Slight reduction hepatic lesions	1
**Gender**	*C. tropicalis*	1	Osteomeyelitis	1	ICD, *Streptococcus sanguis* endocarditis, aorta valve replacement with bioprosthesis	1	Voriconazole + Anidulafungin	1	Cured but complicated with mycotic cerebral aneurysms	1
F: 5 M: 3	*A. fumigatus+ M. genavese*	1	Hepatic abcess	1	persistent pulmonary cavity after radiotherapy for a T1N0M0 lungcarcinoma	1	Itraconasole, L-AMB, Voriconazole	1	Cured from candidemia episode, 4 months later unrelated bacterial sepsis episode	1
					Total parenteral nutrition via Hickmann catheter because of slow transit bowel, intestinal pseudo obstruction, or gastroparesis	3	Anidulafungin and step down to fluconazol	3	Died due to infectious complications 71 or 15 days after initiation of IFN-γ therapy	2
**Placebo treated patients**										
53.0 ± SD19.1	*C. glabrata*	2	Candidaemia	3	Total parenteral nutrition via Hickmann catheter because of slow transit bowel	1	Anidulafungin	2	Cured without further infectious complications	3
**BMI**	*C. tropicalis*	1			HIV with porth-a-cath for venous access	1	Anidulafungin + amphotericin B	1		
18.5 ± SD4.0					construction of ileal conduit urinary diversion (Bricker deviation) because of pT4N2M1 bladder cancer.	1				
**Gender**										
F: 3 M: 0										

F, female; M, male; ICD, implantable cardioverter-defribillator; HIV, human immunodeficiency virus; L-AMB, liposomal amphotericin B; BAL, bronchoalveolar lavage; AML, acute myeloid leukemia.

### Clinical outcome

The three patients in the control group and five out of eight patients treated with rIFN-γ recovered uneventfully from the fungal infection (Table 1). Two patients with invasive aspergillosis that were already admitted to the ICU at the time of treatment died due to infectious complications of severe pulmonary aspergillosis, despite rIFN-γ treatment. The patient with a *Candida* endocarditis, who despite rIFN-γ treatment developed intracerebral mycotic aneurysm, could be discharged from the hospital 93 days after onset of invasive candidiasis.

In all patients treated, rIFN-γ was well tolerated. Five patients reported moderate fever upon administration of rIFN-γ, which responded well to acetaminophen. Two patients developed liver enzyme abnormalities for which tuberculostatic antibiotics and voriconazole were temporarily discontinued, resulting in recovery of the liver enzyme abnormalities while rIFN-γ treatment was continued. No other significant adverse events were observed.

### Effect of rIFN-γ on *ex-vivo* IL-1β and TNFα production

**Figure 2:**
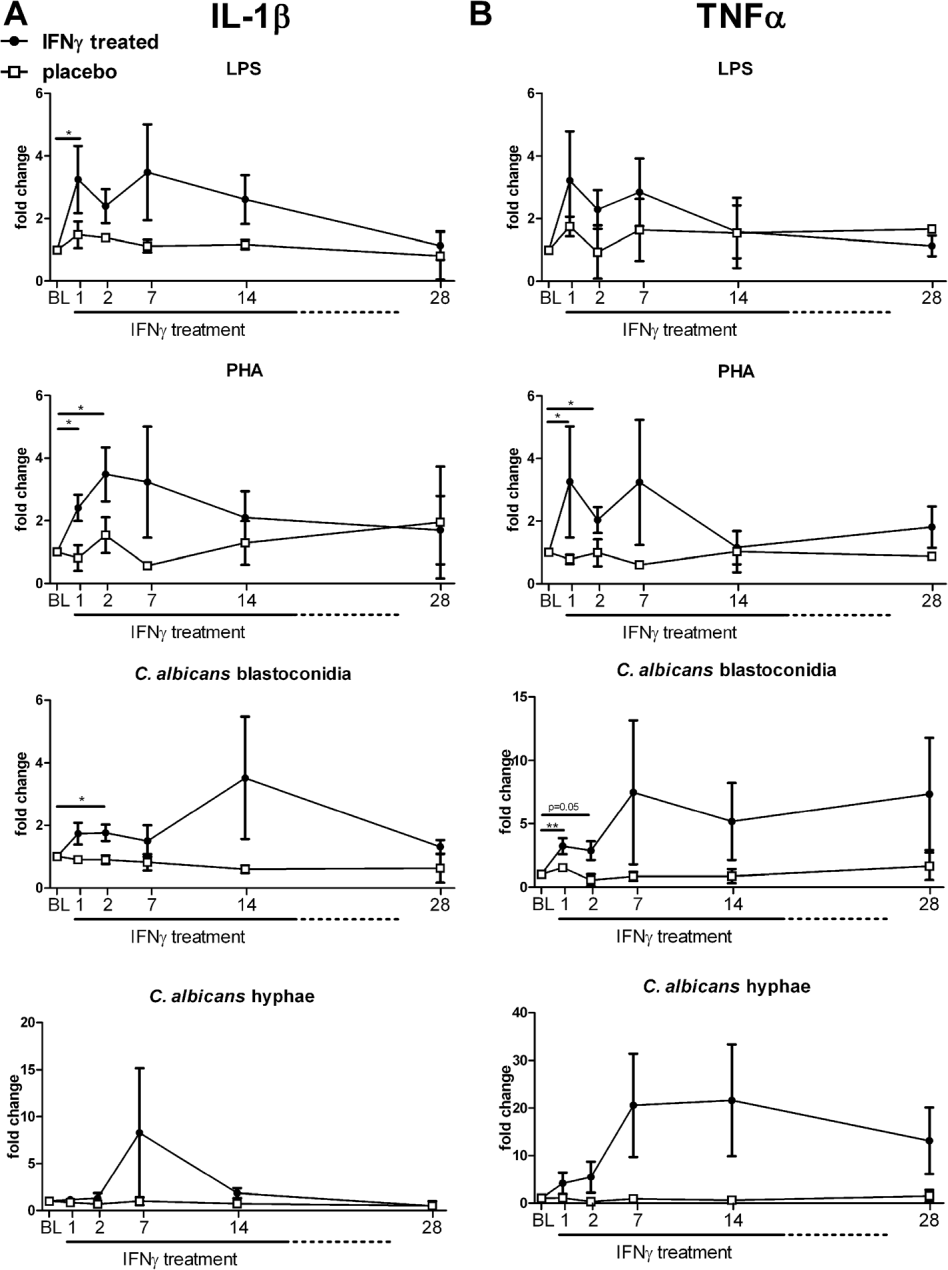
**Effect of rIFN-γ on *ex-vivo *IL-1β and TNFα production.** PBMCs of patients were isolated at baseline and day 1, 2, 7, 14 and 28 after rIFN-γ administration. Isolated PBMCs were stimulated for 24 hours with LPS, PHA, *C. albicans* blastoconidia, or *C. albicans* hyphae. IL-1β **(A)** and TNFα **(B)** concentrations were measured in culture supernatants. Baseline concentrations were used as control and set at 1; subsequent measurements are plotted as the mean relative fold change ± SEM. Significant change from baseline was determined by subjecting the data to Wilcoxons signed rank test. (* = p < 0.05; ** = p < 0.01).

To assess the effect of rIFN-γ on the capacity of PBMCs to produce pro-inflammatory cytokines, cells were isolated and stimulated before, during, and after treatment. We monitored the fold change in cytokine production compared with baseline (before start of treatment). IL-1β and TNFα are pro-inflammatory cytokines of the innate immune system crucial in the induction and maintenance of the anti-fungal immune response [35-40]. Before IFN-γ treatment, inter-patient variability in cytokine production was high (e.g. TNFα median [IQR] concentration after stimulation with LPS was 792 pg/mL [314–2005]). Nevertheless, in all patients an increase in the capacity to induce different cytokines was observed in the first two days after initiation of IFN-γ treatment, independent of their baseline values (group data shown in Figure 2), at subsequent time points only a trend towards increased could be observed. In contrast, the placebo-treated patients IL-1β and TNFα responses over time remained similar to baseline. The response against hyphae of *C. albicans* was highly variable between patients. Some rIFN-γ-treated patients demonstrated a profound increase of TNFα production after treatment (up to 70 fold), whereas other patients showed no relevant change in TNFα production. Cytokine production remained similar in patients in the control group.

### Effect of rIFN-γ on *ex-vivo* IL-17 and IL-22 production

**Figure 3:**
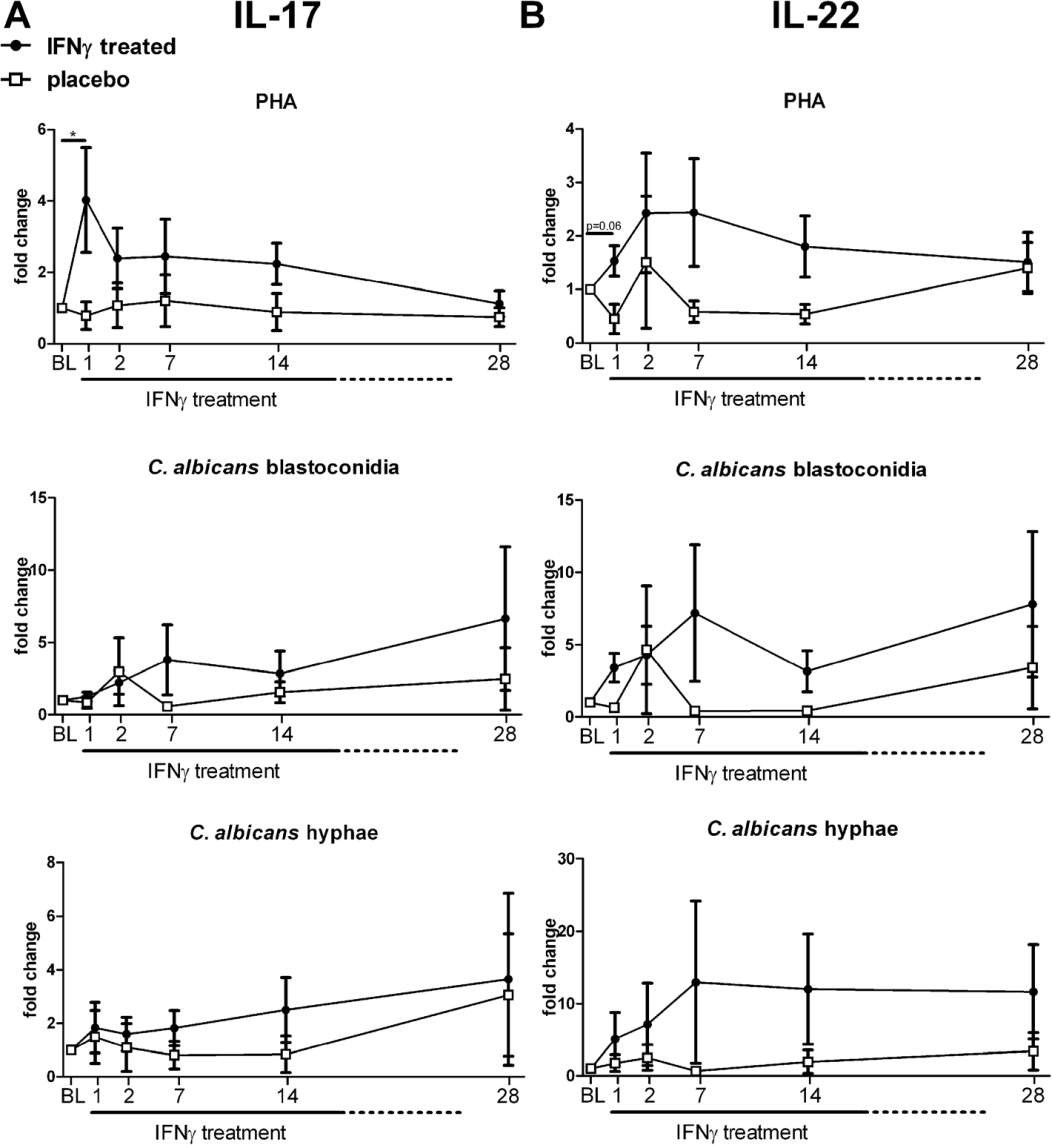
**Effect of rIFN-γ on *ex-vivo *IL-17 and IL-22 production.** PBMCs of patients were isolated at baseline and day 1, 2, 7, 14 and 28 after rIFN-γ administration. Isolated PBMCs were stimulated for 7 days with PHA, *C. albicans* blastoconidia, or *C. albicans* hyphae. IL-17 **(A)** and IL-22 **(B)** concentrations were measured in culture supernatants. Baseline concentrations were used as control and set at 1; subsequent measurements are plotted as the mean relative fold change ± SEM. Significant change from baseline was determined by subjecting the data to Wilcoxons signed rank test. (* = p < 0.05).

Both IL-17 and IL-22 are cytokines that are thought to be protective in the host defence against invasive fungal infections [35,41-45]. PHA-induced IL-17 and IL-22 production was increased 1 day after initiation of rIFN-γ treatment (Figure 3). However, at subsequent time points a trend towards increased IL-17 and IL-22 production was observed, which reverted to baseline levels at day 28. Production of IL-17 and IL-22 upon stimulation with *Candida* blastoconidia was elevated after rIFN-γ treatment in 6 of 8 patients. Hyphae induced IL-17 and IL-22 production was increased in 4 of 8 and 5 of 8 patients respectively. Patients who received placebo therapy did not display a trend towards increased IL-17 or IL-22 production during the course of treatment (group data shown in Figure 3).

### Effect of rIFN-γ on *ex-vivo* IL-10 production

**Figure 4:**
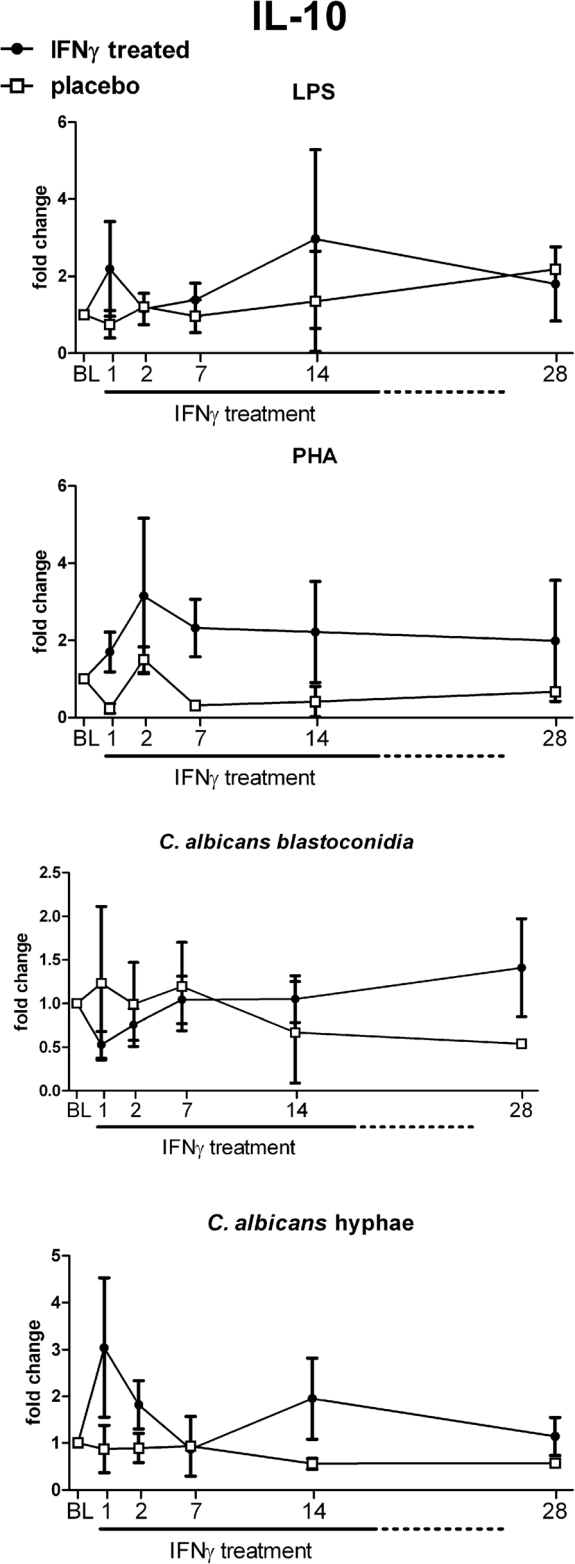
**Effect of rIFN-γ on *ex-vivo *IL-10 production.** PBMCs of patients were isolated at baseline and day 1, 2, 7, 14 and 28 after rIFN-γ administration. Isolated PBMCs were stimulated for 48 hours with LPS, PHA, *C. albicans* blastoconidia, or *C. albicans* hyphae. IL-10 concentrations were measured in culture supernatants. Baseline concentrations were used as control and set at 1; subsequent measurements are plotted as the mean relative fold change ± SEM.

In addition to pro-inflammatory cytokines, the capacity to produce anti-inflammatory cytokines can also influence disease outcome. In particular the anti-inflammatory cytokine IL-10 has been associated with protection against immunopathology during severe infections. IL-10 production in response to stimulation with LPS, PHA and *Candida* was highly variable between patients and did not show a distinct pattern following rIFN-γ treatment (Figure 4). No relevant differences compared to the placebo-treated patients were observed.

### HLA-DR expression

**Figure 5:**
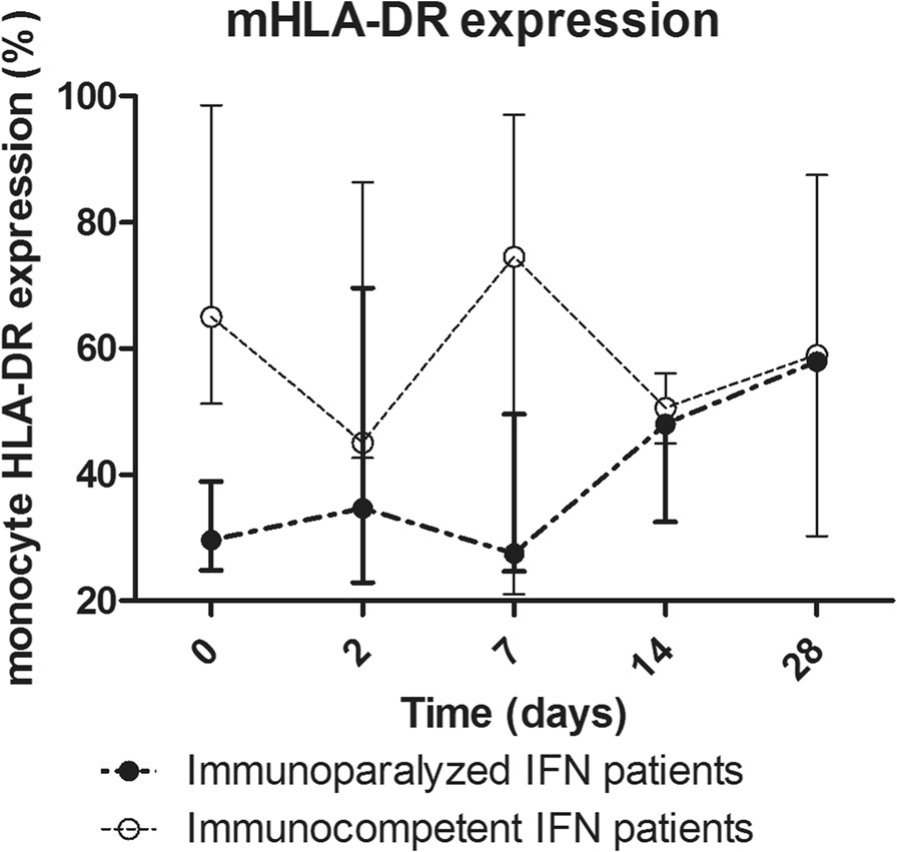
**mHLA-DR expression in rIFN-γ treated patients, divided into immunoparalyzed patients with baseline HLA-DR expression below 50% (solid dots), and without HLA-DR defined immunoparalysis (open dots).** Data are expressed as median [IQR].

The numbers of HLA-DR-positive monocytes, a marker of immunosuppression, varied substantially between patients at baseline (39.05% [27.5-61.6] vs. 90.6 [88.7-92.5] in IFN-γ-treated patients and placebo-treated patients, respectively). Five out of eight IFN-γ treated patients exhibited HLA-DR positive monocyte levels below the “immunoparalysis threshold” of 50% and in these patients, an increase of HLA-DR-positive monocytes after IFN-γ treatment between 10% and 44% was observed which persisted throughout the study period (Figure 5). Patients with a baseline HLA-DR expression higher than 50% did not show a change in expression. The patient with a HLA-DR-expression <50% who did not show increased levels of HLA-DR positive monocyte numbers at any time point, was one of the two patients who died due to infectious complications. No correlation was found between the level of mHLA-DR expression and TNFα production of LPS-stimulated PBMCs. An inverse correlation of baseline mHLA-DR levels with severity of underlying illness and tissue involvement was found (with higher mHLA-DR levels in patients with only impaired physical barriers, e.g. due to indwelling catheters, compared to patients with impaired immune responses, e.g. due to chemotherapy, immune suppressive agents, bone marrow disease; data not shown because this compromises patients anonymity).

### Cell populations

There were no significant changes in the total leukocyte and granulocyte numbers in rIFN-γ-treated patients (Additional file 3: Figure S2A). Monocyte counts significantly increased one week after initiation of rIFN-γ therapy (Additional file 3: Figure S2C) and lymphocyte numbers significantly increased at 2 and 7 days after initiation of rIFN-γ therapy (Additional file 3: Figure S2D), which could be attributed to slight changes in CD4 lymphocytes (Additional file 3: Figure S2E), B-lymphocyte (Additional file 3: Figure S2F) and NK-cell numbers (Additional file 3: Figure S2G) and a significant increase of CD8 lymphocytes (Additional file 3: Figure S2H). No clear changes in leukocyte (subset) counts were observed in placebo-treated patients.

## Discussion and conclusions

While several small clinical trials illustrated the beneficial clinical effects of adjuvant treatment with IFN-y, the proposed immunostimulating effect of IFN-γ as the mechanism of action has not been investigated. In this case series we demonstrate for the first time that adjunctive immunotherapy with rIFN-γ improves the leukocyte immune responses in patients with severe invasive fungal infections. This was primarily reflected by increased *ex-vivo* pro-inflammatory cytokine responses of the innate immune system such as IL-1β or TNFα, as well as an increased production of the T-cell cytokines IL-17 and IL-22, which are known to play an important role in the anti-fungal host defence [35,41-45], and by an increase in HLA-DR expression in mHLA-DR expression in those patients with a low cellular expression as a measure of their immune suppression.

In addition to enhanced *ex-vivo* responses, subtle changes in the leukocyte differentiation were observed following IFN-γ treatment. Although there were no significant differences in total leukocyte numbers after treatment with rIFN-γ, shifts in leukocyte subpopulations such as increased monocyte and lymphocyte counts were apparent. While lymphocyte numbers increased after rIFN-γ therapy, it could not directly be attributed to a specific subset as all of them showed increased values. The most significant increase was that of CD8 cells one week after initiation of rIFN-γ therapy. Monocytes and lymphocytes are known to be crucial cells in the host defence against fungal infections. However, the increase of monocytes and lymphocytes during rIFN-γ therapy was accompanied by slightly decreased circulating granulocyte numbers. It is not known whether this reduction is due to activation and migration into the infected tissue, or whether a true decrease in granulocyte generation was induced by the treatment. Although the decrease in granulocyte numbers was slight, the fact that granulocytes, and especially neutrophils, are crucial in the antifungal host defence warrant careful monitoring of granulocyte numbers during IFN-γ treatment.

Several clinical studies and case reports have previously demonstrated beneficial effects of rIFN-γ in combination with antifungal therapy on outcome of fungal infections (for example in patients with CGD (n = 130) [22,46,47], HIV (n = 173) [23-25], leukaemia (n = 5) [26,27], and transplant patients (n = 7) [28], in a patient with *S. aureus* liver abscess and invasive *C. albicans* infection [48], in a patient with intracerebral aspergillosis [49], in two patients with progressive chronic pulmonary aspergillosis [50], and in two patients with idiopathic CD4 lymphopenia and cryptococcal meningitis [51]). However, in contrast to our study, *ex-vivo* immune responses in these patients were not investigated. Due to the limited number of patients and the very heterogeneous population, we could not assess clinical endpoints, although a mean mortality of 25% in the IFN-γ treated patients lies below the mean 40% estimated in patients with invasive fungal infections [10,52].

To the best of our knowledge, we are the first to describe mHLA-DR expression, a widely used marker of immunosuppression in (bacterial) sepsis patients [53], in patients with invasive fungal infections. In all IFN-γ treated patients who showed baseline mHLA-DR levels below the immunoparalysis threshold of 50% and survived, IFN-γ mediated upregulation of mHLA-DR expression was observed. In agreement with the data presented in this case series, rIFN-γ has been shown to significantly increase numbers of HLA-DR-positive monocytes both in a human preclinical bacterial sepsis model and in septic patients [31,54]. Reduced production of TNFα by leukocytes *ex-vivo* stimulated with LPS has also been shown to be marker of immunoparalysis in sepsis patients. In contrast to our study, mHLA-DR expression and *ex-vivo* TNFα production were found to be highly correlated in bacterial sepsis patients [54,55]. A possible explanation for this discrepancy is that, in contrast with the emerging consensus that immunoparalysis renders patients more vulnerable to opportunistic infections in general [53], different defects in immune defences may be responsible for enhanced susceptibility towards different pathogens.

Based on the apparent inverse correlation of baseline mHLA-DR levels with severity of underlying illness and tissue involvement, mHLA-DR levels seem to reflect disease severity and general immune status, and not specific immune defects per se. Hence, patients with invasive fungal infections and associated impaired anti-fungal immune responses will probably benefit more from immunostimulatory treatment compared to patients with only impaired physical barriers, e.g. due to indwelling catheters and apparent intact anti-fungal immune responses. Biomarkers reflecting the capacity of specific anti-fungal immune defences are required to identify patients who suffer from invasive fungal infections due to impaired cell-mediated immunity. It is important to identify such patients and attempt a tailored immunotherapeutic approach guided by the actual level and type of immunoparalysis of that specific patient. A blood based assay has been described that demonstrates a failure to induce IFN-γ expression in renal transplant patients and differences in IL-10 and TNFα expression [56], which could be promising biomarkers to identify patients who could benefit from adjunctive immunotherapy.

The intracellular mechanism(s) through which the beneficial effects of IFN-γ are mediated remain to be elucidated. Recently it was proposed that IFN-γ exerts its effects at the transcription level [57], while others have demonstrated that IFN-γ reverses tolerance-associated epigenetic modifications [58]. Another possible mechanism involved in the IFN-γ mediated reversal of immunoparalysis is the downregulation of negative TLR regulators such as IRAK-M, a protein that negatively regulates LPS-induced inflammatory responses and contributes to the development of immunoparalysis [59].

Administration of rIFN-γ was tolerated well. Several patients developed a mild fever upon administration, which responded well to acetaminophen treatment. No other side effects were observed. The most important limitation of the present study is the limited number of patients studied. Because the control group consisted of only three patients, no statistical analysis between the treatment and control groups could be performed. However, despite the small sample size, the increase in HLA-DR expression in patients with mHLA-DR expression levels below 50% and the increased *ex-vivo* response of several cytokines that are crucial in antifungal host defence is a promising observation that underlines the potential of immunotherapy. The slow enrolment of patients presenting with candidemia was the main factor contributing to the decision to terminate the phase IIIb *Candida* pilot-study early. With a reported incidence of 2.5-11 per 100,000 persons in Europe [60], and based on previous epidemiological data in our hospital this low enrollment was not expected at the time of the initiation of the study. The much lower incidence of candidemia in the last two years in our hospital is most likely due to a new antibiotic stewardship introduced recently in our hospital, which has reduced the incidence of opportunistic infections. The cut-off value of mHLA-DR expression levels of 50% to distinguish between immunoparalyzed and immunocompetent patients is another limitation of this study, as this is an arbitrary value chosen. We chose this value because it is well below the 99% CI of mHLA-DR values in healthy volunteers [31]. Therefore, patients with mHLA-DR below 50% do have an impaired antigen presenting capacity of their monocytes which we show to be enhanced by IFN-γ therapy. Whether this cut-off value truly represents immunoparalysis, reflected by enhanced susceptibility to secondary infections or reduced capacity to clear opportunistic infections, remains to be investigated. Furthermore, the use of a standardized analysis technique to quantify mHLA-DR, such as the Quantibrite method, is preferable, because it facilitates an objective comparison of mHLA-DR expression levels between studies and aids in the definitive establishment of a cut-off value to identify immunoparalyzed patients. Larger studies are required to confirm the data obtained here. To do so, multicentre studies should be facilitated in order to fully explore the potential of IFN-γ immunotherapy.

Our data indicate that adjunctive immunotherapy with rIFN-γ in patients with invasive fungal infections partially restores cell-mediated immunity. This suggests that IFN-γ treatment enhances anti-fungal immunity and larger studies are warranted to validate the findings reported here and to assess the impact of IFN-γ treatment on clinical outcome. Biomarkers of impaired anti-fungal immunity should be further investigated in order to identify patients who will benefit most from immunostimulatory therapy.

## Competing interests

None of the authors have any competing interests regarding this study. The support for the immunological assessments in this study was provided by an unrestricted grant from BioMérieux.

## Authors’ contributions

CD, PP, AP, BJK and MN conceived and designed the study. CD, JL and CBR screened and included patients. MG and FF carried out in-vitro experiments. CD, MG, JL, FV and MK analysed the data. MG, JL, FP. MK, PP, GM, AP and MN participated in the data interpretation. CD, MG, JL wrote the manuscript draft. MG, JL, MK, PP, BJK and MN contributed to writing the final manuscript. All authors read and approved the final manuscript.

## Pre-publication history

The pre-publication history for this paper can be accessed here:

http://www.biomedcentral.com/1471-2334/14/166/prepub

## Supplementary Material

Additional file 1 Online Supplement Flow cytometric analysis of mHLA-DR expression and lymphocyte subset counts. Click here for file

Additional file 2: Figure S1 Representative flow diagram of monocyte HLA-DR measurements. Heparin blood was first analysed on forward- and side scatter to exclude cell debris and erythrocytes (A). Subsequently, CD45^+^ cells were selected (B) and within the CD45^+^ fraction was gated for CD14^+^ cells (C). The CD45^+^ CD14^+^ cells (D) were analysed for the percentage of HLA-DR positivity (E). Click here for file

Additional file 3: Figure S2 Changes in immune cell populations. Total leukocyte numbers (A) and numbers of granulocytes (B), monocytes (C) and lymphocytes (D) measured in peripheral blood. Numbers of CD4 lymphocytes (E), B-lymphocytes (F), CD8 lymphocytes (G) and NK cells (H) within the lymphocyte population were quantified using flowcytytometry. Click here for file
