# Supplementary material for: Interferon-gamma as adjunctive immunotherapy for invasive fungal infections: a case series
Source: BMC Infect Dis. 2014 Mar 26;14:166. doi: 10.1186/1471-2334-14-166 (PMC3987054; doi:10.1186/1471-2334-14-166)
Supplement: Additional file 1 — Online Supplement Flow cytometric analysis of mHLA-DR expression and lymphocyte subset counts. [file 1471-2334-14-166-S1.doc]

ONLINE SUPPLEMENT

**Flow cytometric analysis of mHLA-DR expression and lymphocyte subset counts**

To ascertain that expression levels did not change due to a delay between withdrawal and analysis, we performed separate experiments on 5 different blood samples. Expression was determined immediately after withdrawal and after 24 hours storage at 4°C. When samples were immediately stored at 4°C after withdrawal and analyzed within 24 hours, we did not observe significant differences in % or MFI compared with samples that were immediately analyzed after withdrawal. Therefore, analysis was performed within 24 hours after immediate storage at 4°C. After withdrawal, 100 μl blood was incubated with the following fluorochrome-conjugated monoclonal antibodies, for 15 minutes protected from light at 4°C. After erythrocyte lysis (NH_4_CL: 180 mL + 20 mL lysis stock dilution [BD Pharm-Lyse, BectonDickinson]), cells were washed three times in PBS and monocytes and lymphocytes were identified in a 8-color immunophenotyping (NAVIOS flow cytometer, Beckman Coulter, Miami). Monocytes and lymphocytes were identified by forward and side scatter and by cell-specific binding. The following monoclonal antibodies were used for monocyte HLA-DR analysis: HLA-DR-PE (Immu-357), CD14-ECD (RMO52), CD45-KO (J33). Lymphocyte subpopulations were identified by gating on the lymphocyte population in the CD45/SS plot followed by a gating on CD3-APC (UCHT1),  CD4-PECy5.5 (13B8.2),  CD8-APCAlexa700 (B9.11), CD19-APCAlexa750 (HD37)  and CD56-PECy7 (N901) to determine the helper T cells, cytotoxic T cells, B cells and NK cells within the lymphocyte gate (all MoAbs were obtained from Beckman Coulter, Marseille, France).
