# Supplementary figures and images for: Interferon-gamma as adjunctive immunotherapy for invasive fungal infections: a case series
Source: BMC Infect Dis. 2014 Mar 26;14:166. doi: 10.1186/1471-2334-14-166 (PMC3987054; doi:10.1186/1471-2334-14-166)

## Slide 1
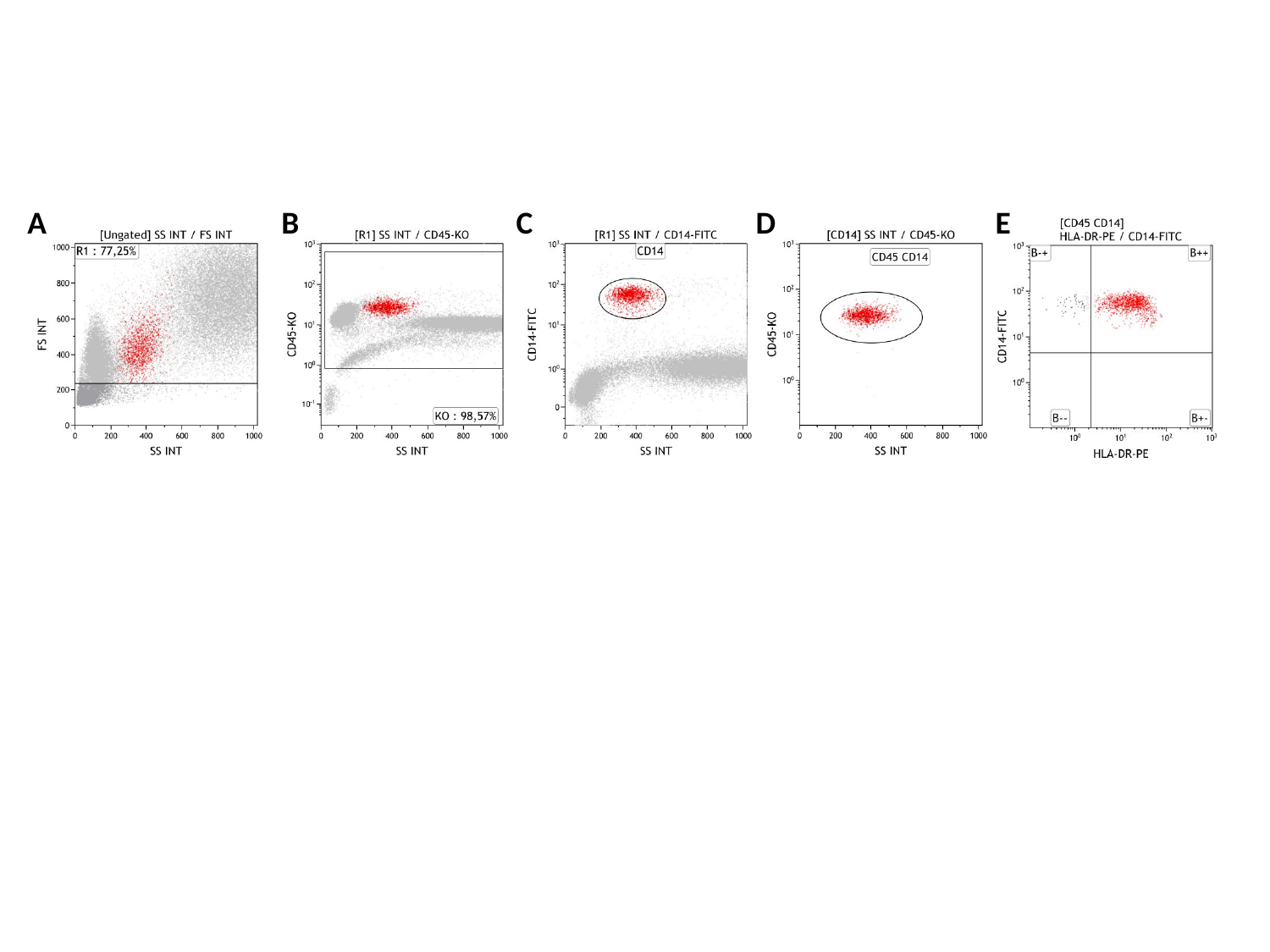

A		B	 C	 	 D		 E

Supplement: Additional file 2: Figure S1 — Representative flow diagram of monocyte HLA-DR measurements. Heparin blood was first analysed on forward- and side scatter to exclude cell debris and erythrocytes (A). Subsequently, CD45+ cells were selected (B) and within the CD45+ fraction was gated for CD14+ cells (C). The CD45+ CD14+ cells (D) were analysed for the percentage of HLA-DR positivity (E). [file 1471-2334-14-166-S2.ppt]

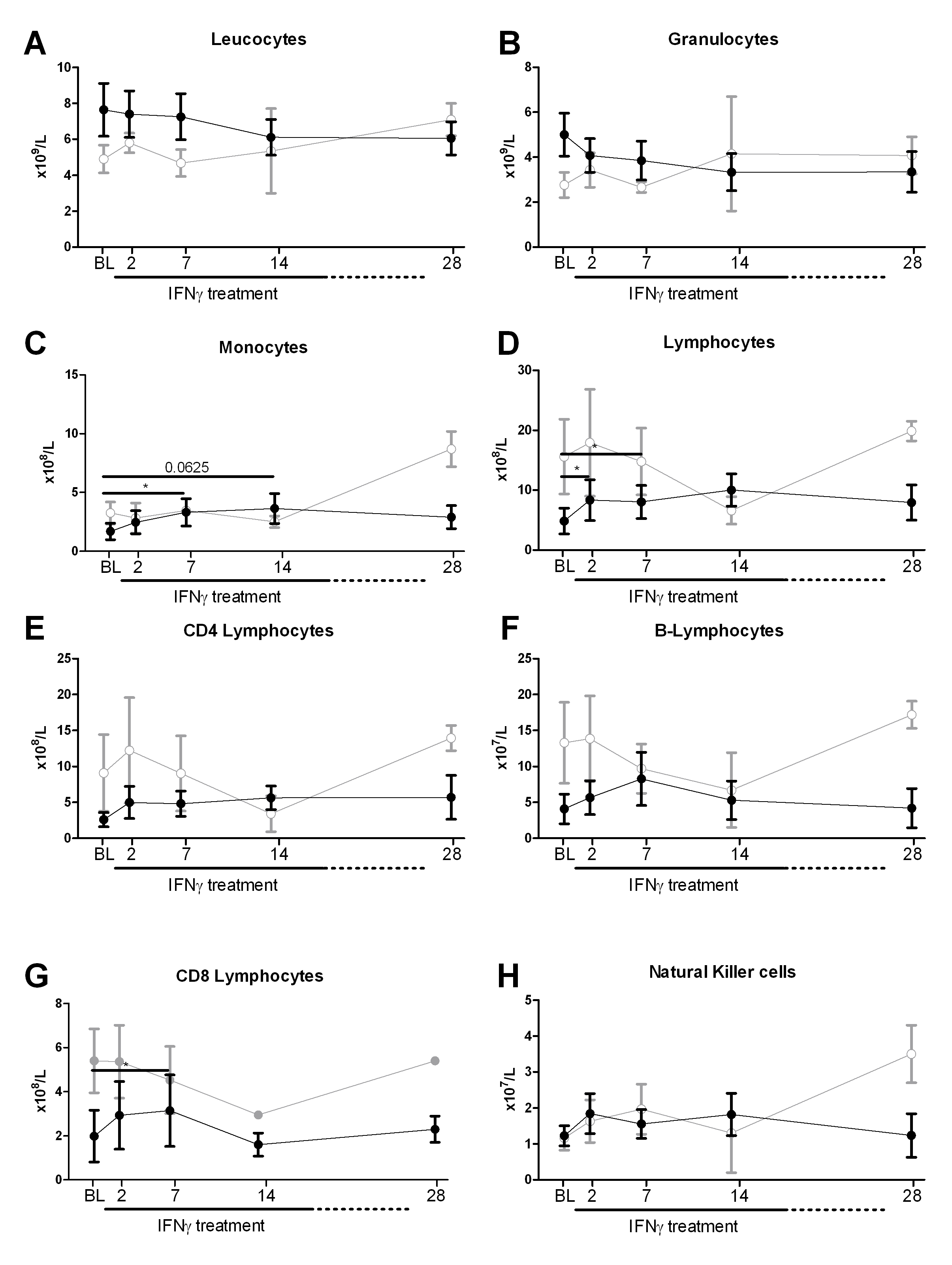

Supplement: Additional file 3: Figure S2 — Changes in immune cell populations. Total leukocyte numbers (A) and numbers of granulocytes (B), monocytes (C) and lymphocytes (D) measured in peripheral blood. Numbers of CD4 lymphocytes (E), B-lymphocytes (F), CD8 lymphocytes (G) and NK cells (H) within the lymphocyte population were quantified using flowcytytometry. [file 1471-2334-14-166-S3.tiff]
